# Histone Deacetylase Inhibitors Dose-Dependently Switch Neutrophil Death from NETosis to Apoptosis

**DOI:** 10.3390/biom9050184

**Published:** 2019-05-11

**Authors:** Hussein J. Hamam, Nades Palaniyar

**Affiliations:** 1 Program in Translational Medicine, Peter Gilgan Centre for Research and Learning, The Hospital for Sick Children, Toronto, ON M5G 0A4, Canada; hussein.hamam@sickkids.ca; 2 Department of Laboratory Medicine and Pathobiology, Faculty of Medicine, University of Toronto, Toronto, ON M5S 1A8, Canada; 3 Institute of Medical Sciences, Faculty of Medicine, University of Toronto, Toronto, ON M5S 1A8, Canada

**Keywords:** neutrophils, neutrophil extracellular trap formation, histone acetylation, histone decondensation, histone deacetylase inhibitors, cytotoxicity, apoptosis

## Abstract

Acetylation is an important post translational modification of histone that plays a role in regulation of physiological and pathological process in the body. We have recently shown that the inhibition of histone deacetylases (HDAC) by low concentrations of HDAC inhibitors (HDACis), belinostat (up to 0.25 µM) and panobinostat (up to 0.04 µM) promote histone acetylation (e.g., AcH4) and neutrophil extracellular trap formation (NETosis). Clinical use of belinostat and panobinostat often leads to neutropenia and the in vivo concentrations vary with time and tissue locations. However, the effects of different concentrations of these HDACis on neutrophil death are not fully understood. We considered that increasing concentrations of belinostat and panobinostat could alter the type of neutrophil death. To test this hypothesis, we treated human neutrophils with belinostat and panobinostat in the presence or absence of agonists that promote NOX-dependent NETosis (phorbol myristate acetate or lipopolysaccharide from *Escherichia coli* 0128) and NOX-independent NETosis (calcium ionophores A23187 or ionomycin from *Streptomyces conglobatus*). Increasing concentrations of HDACis induced histone acetylation in a dose-dependent manner. ROS analyses showed that increasing concentrations of HDACis, increased the degree of NOX-derived ROS production. Higher levels (>1 µM belinostat and >0.2 µM panobinostat) of AcH4 resulted in a significant inhibition of spontaneous as well as the NOX-dependent and -independent NETosis. By contrast, the degree of neutrophil apoptosis significantly increased, particularly in non-activated cells. Collectively, this study establishes that increasing concentrations of belinostat and panobinostat initially increases NETosis but subsequently reduces NETosis or switches the form of cell death to apoptosis. This new information indicates that belinostat and panobinostat can induce different types of neutrophil death and may induce neutropenia and regulate inflammation at different concentrations.

## 1. Introduction

Neutrophils play a crucial role in the innate immune response as they are the first to encounter and neutralize invaders [1,2]. They make ~60–70% of all white blood cells and use different strategies to fight pathogens, including phagocytosis, oxidative burst and neutrophil extracellular trap (NET) formation [3,4]. Numerous studies have demonstrated that when neutrophils interact with certain inflammatory stimuli (e.g., phorbol 12-myristate 13-acetate (PMA), lipopolysaccharide (LPS), A23187, ionomycin), they undergo a unique form of programmed cell death that results in releasing decondensed chromatin coated with cytotoxic peptides in a form of web-like structures called NETs [5,6]. It is well-known that during NET formation (NETosis), neutrophils generate high levels of reactive oxygen species (ROS) that activate specific kinase pathways, which results in promoter melting, transcriptional firing, chromatin decondensation and finally, NET release [7,8,9]. There are currently two major pathways that explain how NETs are formed: NOX-dependent pathway which requires high cytosolic ROS levels and NOX-independent pathways that depends on mitochondrial ROS (mROS) production [6]. Also, some known granular enzymes, such as myeloperoxidase (MPO) and neutrophil elastase, cover the decondensed chromatin during the final stages of NET formation [7]. 

Lately, attention has been directed towards the relevance of histone modifications in delineating the molecular mechanism of NET formation. Studies reported that citrullination of histones has the potential to decondense chromatin during NET formation [7,10]. It is well known that increased calcium influx activates peptidylarginine deiminase 4 (PAD4), which converts the arginine present on histones (e.g., H3) into citrulline. Citrullination of histone H3 (CitH3) results in histone relaxation and was considered as a hallmark of NOX-independent NETosis [11]. We have recently shown that another epigenetic modification—histone acetylation (e.g., acetylated histone H4, AcH4)—promotes both types of NET formation [12]. Due to the loss of positively charged lysine, AcH4 results in increased levels of histone decondensation and gene transcription which eventually promoted NETosis by ~20% without altering intracellular ROS production by either NOX or mitochondria. To achieve increased levels of AcH4, histone deacetylase complex (HDAC) inhibitors within their half maximal inhibitory concentrations (IC50) were used because primary neutrophils express 18 different HDACs [13,14]. These inhibitors (HDACis) can act on most HDACs in the form of pan-deacetylase (pan-DAC) inhibitors and three of them are approved for clinical use, including belinostat and panobinostat. In fact, some clinical studies have shown some adverse effects of using HDACis in treating diseases. For example, a phase 2 study showed that treating myelodysplastic syndrome with belinostat resulted in 19 and 48 % of patients with grade 2 and 3/4 neutropenia, respectively [15]. Also, despite that belinostat and panobinostat are used in a large range of concentrations depending on their applications, the relevance of these HDAC is in inducing different cytotoxic effects in neutrophils is not yet known [16]. Therefore, treating neutrophils with increasing concentrations of either belinostat or panobinostat could result in neutrophil apoptosis or NET formation.

We hypothesized that treating neutrophils with increasing concentrations of HDACis would promote apoptosis and suppress NETosis. To examine this, we utilized belinostat and panobinostat on primary neutrophils of healthy donors and tested histone acetylation, ROS production and NETosis at baseline and with the use of agonists for NOX-dependent and -independent pathways. Our studies show that increasing concentrations of HDACis promote histone acetylation while suppressing spontaneous NET formation in a dose-dependent manner. Also, HDACis significantly inhibits NETosis in the presence of NETotic agonists. Nevertheless, a significant increase in apoptosis was accompanied with the inhibition of NET formation, particularly in non-activated cells. In addition, HDACis significantly increased the cytosolic but not mitochondrial, ROS levels, which could promote both apoptosis and NETosis. Therefore, the data presented in this study show that HDACis have a biphasic effect on NET formation. For the first time, we show direct evidence that belinostat and panobinostat have both NETotic and apoptotic effects on neutrophils. These findings would be useful in improving our understanding of the molecular mechanism of NETosis and clinical use of HDAC inhibitors for treating conditions including autoimmune diseases (e.g., lupus) and cancers [17,18,19,20].

## 2. Materials and Methods

### 2.1. Research Ethics Board Approval

The study protocol for using human blood samples was approved by the ethics committee of The Hospital for Sick Children (No. 1000020217). The ethics committee guidelines were followed while performing all procedures, including healthy human volunteer recruitment for blood donation. All the volunteers participated in this study signed informed consents prior to the blood donation.

### 2.2. Human Peripheral Blood Neutrophil Isolation

In this study, neutrophils from healthy male donors were used. However, data were excluded if donors had eosinophils or either too low or too high neutrophil count. A volume of 40–60 mL of peripheral blood, which usually yields 1–1.5 million neutrophils per millilitre of blood, was drawn from healthy donors into K2-EDTA (ethylenediaminetetraacetic acid) blood collection tubes (Becton, Dickinson and Co., Franklin Lakes, NJ, USA) at the nursing station of the hospital. Neutrophils were purified from peripheral blood by using PolymorphPrep^TM^ (Axis-Shield, Oslo, Norway) by following the manufacturer’s instructions but with minor modifications. Blood was layered over PolymorphPrep^TM^ solution in a 1:1 ratio and was then centrifuged at 600× *g* for 35 min without any brakes. Then, the polymorphonuclear neutrophil layer was collected and washed with 0.425% (*w/v*) NaCl with 10 mM HEPES (4-(2-hydroxyethyl)-1-piperazineethanesulfonic acid) to eliminate residues of PolymorphPrep^TM^. A hypotonic solution (0.2% (*w/v*) NaCl) was used twice for 30 s to purify neutrophils from the residual red blood cells (RBCs). To obtain isotonic condition, neutrophils were immediately mixed with an equal volume of 1.6% (*w/v*) NaCl solution with 20 mM HEPES buffer. Next, the cells were washed twice to eliminate RBC debris and soluble components. Neutrophils were re-suspended by using Roswell Park Memorial Institute (RPMI) 1640 medium (Invitrogen, Carlsbad, CA, USA) supplemented with 10 mM HEPES buffer. A haemocytometer was used to quantify cell density and Cytospin preparations were used to check the purity of the neutrophils. Neutrophil preparations with >95–98% were used in all of the experiments.

### 2.3. Sytox Green NETosis Assay

To estimate NETosis kinetics, we used a cell-impermeable DNA binding dye called Sytox Green (Life Technologies, Carlsbad, CA, USA). A concentration of 5 µM Sytox Green were added in 100 µL media (RPMI 1640 medium supplemented with 10 mM HEPES) containing 50,000 neutrophils and then seeded into 96-well black clear-bottom plates. A volume of 5 µL of HDACis (final concentrations: 0.5, 1, 2, 4, 5, 10, 20 and 40 µM belinostat (PXD-101, Selleckchem); 0.08, 0.2, 0.4, 0.8, 1.6, 3.2 and 6.4 µM panobinostat (LBH589, Selleckchem) was added to respective wells with controls (RMPI + neutrophils only) for 30 min at 37 °C and 5% (*v/v*) CO_2_. A volume of 5 µL agonists (final concentrations: 25 nM PMA; 4 μM A23187; 5 μg/mL LPS (from *E. coli* 0128); 5 μM ionomycin, (unless otherwise stated)) was then added and placed at 37 °C and 5% (*v*/*v*) CO_2_ incubator. By using a fluorescence plate reader, the Sytox Green fluorescence intensities were measured every 60 min for up to 4 h (504 nm excitation, 523 nm emission, POLARstar OMEGA, BMG Labtech, Guelph, ON, Canada). Finally, the NETotic index (% of Sytox Green accessible total DNA) was calculated by subtracting the baseline green fluorescence at time 0-min from the fluorescence at each time point and was then divided by the fluorescence values of 100% NET formation obtained by lysing the cells with 0.5% (*v*/*v*) Triton X-100 (240 min time point).

### 2.4. DHR123 and MitoSOX Plate Reader Assays (NOX- and Mitochondrial-Mediated ROS Analyses)

A concentration of 20 μM dihydrorhodamine 123 (DHR123, Thermo Fisher Scientific, Waltham, MA, USA) was added in media containing 100,000 neutrophils and were then incubated at 37 °C and 5% (*v*/*v*) CO_2_ incubator for 15 min. Neutrophils were centrifuged for 10 min (400× *g*; with nine acceleration and nine deceleration ramps) and were washed with an equal amount of media. A volume of 100 μL of the neutrophil-containing solution was seeded into 96-well black clear-bottom plates and HDACis (belinostat and panobinostat) were added to respective wells with controls (RMPI + neutrophils only) for 30 min at 37 °C and 5% (*v*/*v*) CO_2_. A volume of 5 µL agonists (final concentrations: 25 nM PMA; 4 μM A23187; 5 μg/mL LPS from *E. coli* 0128; 5 μM ionomycin) were then added and incubated at 37 °C and 5% (*v*/*v*) CO_2_. A fluorescence plate reader was used to measure the fluorescence (507 nm excitation, 529 nm emission, POLARstar OMEGA, BMG Labtech) every 10 min in the first 30 min and then every 30 min up until 90 min. To calculate the relative fluorescence units (RFU), the fluorescence at each time point was subtracted from the baseline fluorescence at time 0-min.

The MitoSOX Red (Thermo Fisher Scientific) plate reader assays were performed similarly to the DHR123 assay with the following exceptions: 5 μM MitoSOX red were incubated at 37 °C and 5% (*v*/*v*) CO_2_ incubator for 15 min (no washing) and measured by fluorescence plate reader (510 nm excitation, 580 nm emission).

### 2.5. Fluorescence Confocal Imaging

A volume of 100 µL of media containing 100,000 neutrophils were seeded into 12-well chamber slides. A volume of 5 µL of HDACis (0.5, 2, 5 and 20 µM belinostat; 0.08, 0.4, 0.8 and 3.2 µM panobinostat) and/or NETotic agonists (25 nM PMA; 4 μM A23187; 5 μg/mL LPS from *E. coli* 0128; 5 μM ionomycin) were then added to respective wells with controls (RMPI + neutrophils only) and incubated for 120 min at 37 °C and 5% (*v*/*v*) CO_2_. As a positive control for apoptosis, neutrophils were treated with ultraviolet (UV) irradiation (0.24 J/cm^2^). To fix neutrophils and NETs, paraformaldehyde (4%, *w*/*v*) was used for 30 min and cells were then washed and permeabilized with 0.1% Triton-X 100 for 15 min at room temperature. Unspecific antigens were then blocked by using bovine serum albumin (BSA) (5%, *w*/*v*) for 60 min at room temperature. The cells were washed with phosphate-buffered saline (PBS) and incubated with primary antibodies: mouse anti- myeloperoxidase (MPO) antibody (ab25989, Abcam; 1:500 dilution) was used for staining MPO (with secondary antibody conjugated with a green fluorescence Alexa Fluor 488 dye; 1:1000 dilution; Thermo Fisher Scientific), while rabbit anti-CitH3 antibody (ab5103, Abcam; 1:500 dilution) or rabbit anti-histone H4 (acetyl K5) antibody (ab51997, Abcam, 1:1000) was used for detecting the presence of CitH3 or acetylated H4K5 (H4K5ac), respectively (with secondary antibody conjugated with a far-red fluorescence dye Alexa Fluor 647; 1:1000dilution; Thermo Fisher Scientific). The 4′,6-diamidino-2-phenylindole (DAPI) (1:100 dilution) was used to stain DNA. After treating with the secondary antibody, slides were washed and mounted by glass coverslips (Fisher Scientific) with anti-fade fluorescent mounting medium (Dako, Carpinteria, CA, USA). To acquire images, Olympus IX81 inverted fluorescence microscope was used with a Hamamatsu C9100-13 back-thinned EM-CCD camera and Yokogawa CSU × 1 spinning disk confocal scan head (Olympus Canada Inc., Richmond Hill, ON, Canada) with Spectral Aurora Borealis upgrade, four separate diode-pumped solid-state laser lines (405, 491, 561 and 642 nm; Spectral Applied Research, Richmond Hill, ON, Canada). The images were taken at 20 × /0.75 and 40 × /0.95 magnification and processed by Volocity software (version 6.3, Cell Imaging Perkin-Elmer; Quorum Technologies Inc., Puslinch, ON, Canada).

### 2.6. Western Blotting

A volume of 5 µL of HDACis (0.5, 2, 5 and 20 µM belinostat; 0.08, 0.8 and 3.2 µM panobinostat), NETotic agonists (25 nM PMA; 4 μM A23187; 5 μg/mL LPS from *E. coli* 0128; 5 μM ionomycin) and/or ultraviolet (UV) irradiation (0.24 J/cm^2^) were added to tubes containing 1 × 10^6^ cells and incubated for 90 min at 37 °C and 5% (*v*/*v*) CO_2_. After being cooled on ice for 10 min, radioimmunoprecipitation assay (RIPA) lysis buffer (Millipore, Etobicoke, ON, Canada) containing 1 mM phenylmethylsulfonyl fluoride (PMSF), 1 mM sodium orthovanadate, 1 mM sodium fluoride, 1 mg/mL aprotinin, 1 mg/mL leupeptin, 1 mg/mL pepstatin, protease inhibitor cocktail tablet per 5 mL (Roche Diagnostics, Laval, QC, Canada), DNase I (Invitrogen) and a phosphatase inhibitor cocktail tablet per 10 mL (Roche) was used to lyse neutrophils. Samples were vortexed for 10 s to mix the solution and incubated for 30 min at 37 °C and 5% (*v*/*v*) CO_2_ and then sonicated three times for 30 s intervals. The bicinchoninic acid (BCA) protein assay kit (Thermo Fisher Scientific) was used to ensure the same amount of total protein of each sample was used. A reducing sample loading buffer (20% (*v*/*v*) glycerol, 2% (*v*/*v*) beta-mercaptoethanol, 4% (*w*/*v*) sodium dodecyl sulfate (SDS), 0.130 M Tris, bromophenol blue (1 mg/100 mL), pH 6.8) was added to each sample and were then heated at 99 °C for 5 min prior to loading into the SDS-PAGE (polyacrylamide gel electrophoresis). Samples were run on a 4–20% Mini-PROTEAN^®^ TGX™ precast protein gels (Bio-RAD Laboratories, Mississauga, ON, Canada) at 120 V for 60 min to separate protein by size. Wet transfer was performed to transfer proteins to nitrocellulose membranes. Unspecific binding on the membrane were blocked with 5% (*w*/*v*) BSA in TBST (Tris-buffered saline with 0.1% (*v*/*v*) Tween 20) for 1 h at room temperature. All primary antibodies were dissolved in 1% (*w*/*v*) BSA in 0.1% (*v*/*v*) TBST and incubated with membranes overnight at 4 °C followed by three washes with 0.1% PBST for 30 min. Primary antibodies used were: anti-GADPH (2118, Cell Signalling Technology, Whitby, ON, Canada) rabbit mAb at 1:1000; anti-histone H4K5ac (ab51997, Abcam) rabbit mAb at 1:1000; anti-cleaved caspase-3 (Asp175, Cell Signalling) rabbit mAb at 1:500. Then, the membranes were incubated in the secondary antibody solution (1% (*w*/*v*) BSA in 0.1% (*v*/*v*) TBST) for 60 min and then washed three times with 0.1% (*v*/*v*) PBST for 30 min. The secondary antibody used was: anti-rabbit IgG-HRP (Cell Signalling) at 1:2000. Enhanced chemiluminescent reagents were used to determine the protein intensity and the blots were imaged in Li-Cor Odyssey FC Imaging System and densitometry analysis of the images performed using Image Studio software (LI-COR Biotechnology, Lincoln, NE, USA). The H4K5ac and cleaved caspase 3 protein bands were normalized to the GAPDH bands.

### 2.7. Statistical Analyses

All data are presented as mean ± standard error of the mean (SEM) in line graphs. A two-way analysis of variance (ANOVA) with Dunnett test was performed to analyse line graphs and box plots. In box plots, each data point is presented. The mean is indicated with “+” and the full data spread is indicated with lines and boxes are marked with median and upper and lower interquartile ranges. Best fit linear regression analysis was performed and the equations, r^2^ values and *p*-values are indicated on each panel. The statistical analyses by One-way ANOVA with a Dunnett or Tukey test, where appropriate, were performed by using Prism GraphPad statistical analysis software (Version 8.0.2 for Windows, San Diego, CA, USA). A Dunnett post-test was used for comparing a fixed value of 1 with respective treatment conditions. A mean difference with a *p*-value of ≤0.05 was considered to be statistically significant.

## 3. Results

### 3.1. Increasing Concentrations of HDAC Inhibitors Increase Histone Acetylation

**Figure 1:**
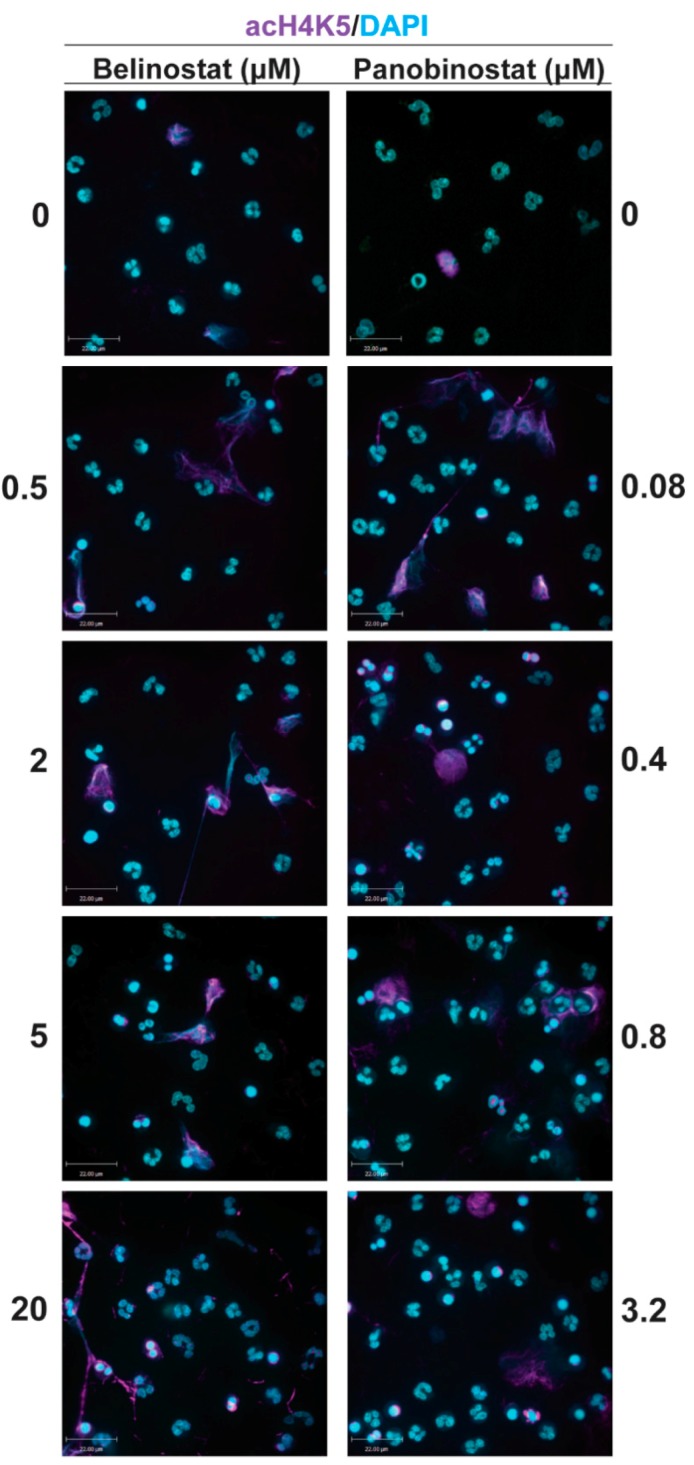
Confocal microscopy images showing histone deacetylase (HDAC) inhibitors promote histone acetylation. Neutrophils were treated with negative control (Roswell Park Memorial Institute (RPMI) media) or HDACis (0.5, 2, 5 and 20 µM belinostat; 0.08, 0.4, 0.8 and 3.2 µM panobinostat) for 120 min. Cells were then imaged after being fixed and immunostained for histone acetylation (H4K5ac) and DNA (4′,6-diamidino-2-phenylindole, DAPI). Neutrophils treated with RPMI show typical polymorphonuclear morphology of neutrophils. When treated with HDACis, neutrophils show a further increase in histone acetylation. Blue, DAPI staining for DNA; Magenta, H4K5ac. Scale bar, 14 µm. *n* = 3. See Supplementary Figure S1 for colocalization data between DAPI and AcH4.

We have recently shown than neutrophils treated with lower concentrations of HDACis lead to increased levels of histone acetylation, chromatin relaxation and NETosis [12,21,22]. Therefore, we questioned whether increasing concentrations of HDACis have the potential to further promote histone acetylation in neutrophils. To check this point, we have performed immunofluorescence assays by using primary neutrophils that were treated with pan-HDACis, belinostat and panobinostat and then stained against DAPI and H4K5ac (AcH4). Immunofluorescence images showed intact neutrophils that were characterized by multi-lobulated nuclei and minor staining for AcH4 (magenta; Anti-H4K5ac) when they were treated with RPMI (negative control; Figure 1). Upon treating the cells with belinostat or panobinostat, we observed increased levels of histone acetylation in a dose-dependent manner, compared to the control. By examining the immunofluorescence images, it was noticeable that AcH4 was present in the nuclei of dying neutrophils and chromatin. To verify this point, we quantified our immunofluorescent images and checked for the colocalization between DAPI and AcH4. The Manders’ overlap coefficients were similar between intact, NETotic and apoptotic cells in all experimental conditions, suggesting that AcH4 overlapped with chromatin (DAPI; Supplementary Figure S1).

**Figure 2:**
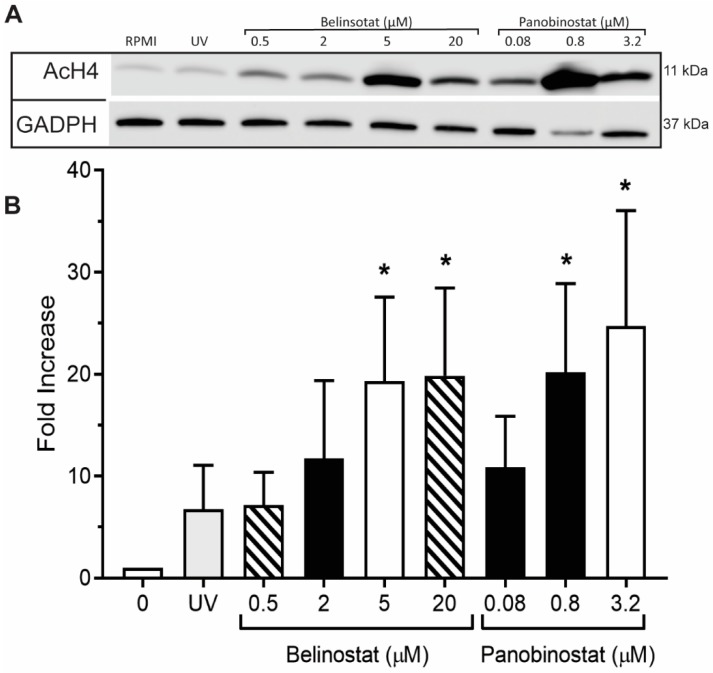
Western blots confirm that HDAC inhibitors induce histone acetylation. (**A**) Neutrophils were treated with RPMI (negative control) or HDAC inhibitors (0.5, 2, 5 and 20 µM belinostat; 0.08, 0.8 and 3.2 µM panobinostat) for 90 min. Equal amounts of lysates from each condition were separated by polyacrylamide gels, transferred onto membranes and specific proteins were immunodetected (GADPH for loading control and H4K5ac for histone acetylation). (**B**) The densitometry analyses show increased histone acetylation when neutrophils are treated with HDAC inhibitors, compared to their corresponding controls. The values were normalized to the negative control values in each experiment. All data are presented as mean ± SEM; *n* = 3; *, *p* < 0.05 compared to respective controls. See Supplementary Figure S2 for the full Western blot.

To confirm the results of our immunofluorescence imaging, we performed Western blots and checked for H4K5ac (AcH4). Results showed negligible levels of histone acetylation for neutrophils treated with RPMI (Figure 2; see Supplementary Figure S2 for uncropped Western blots). However, the immunoblot analysis showed a significant dose-dependent increase in AcH4 levels when cells were treated with HDACis, compared to the control. 

### 3.2. Higher Concentrations of HDAC Inhibitors Suppress Baseline NETosis

**Figure 3:**
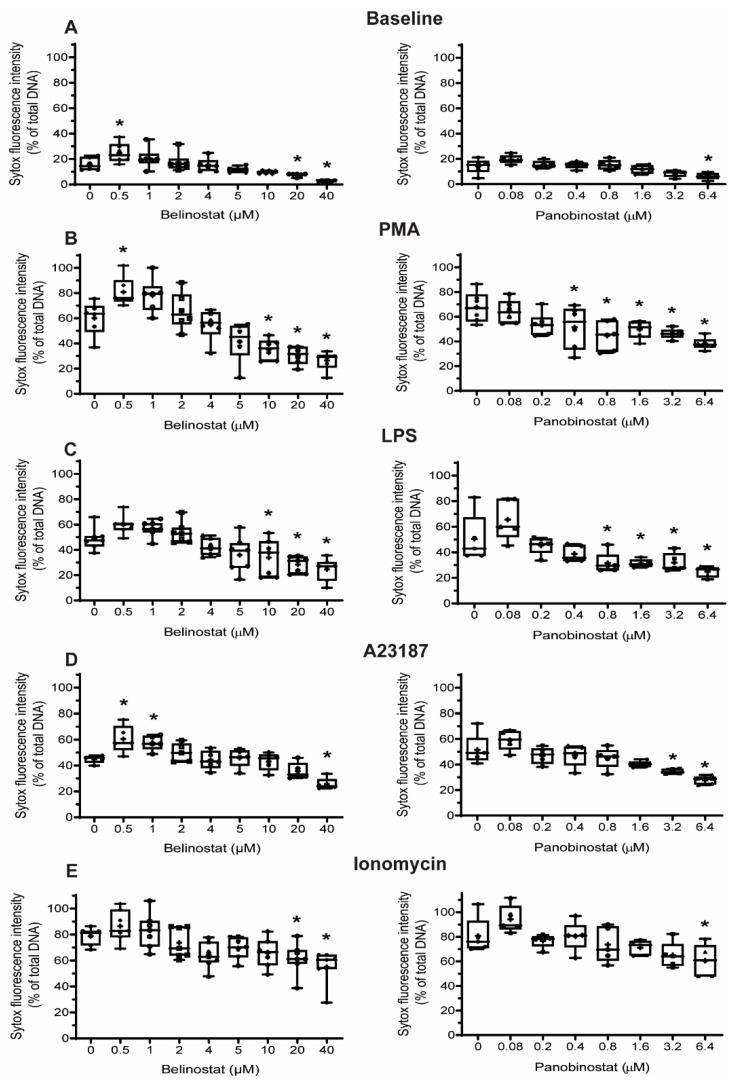
Sytox Green assays suggest that belinostat and panobinostat inhibit baseline NETosis as well as both NOX-dependent and -independent NETosis. Neutrophils were treated with HDACis and/or NETotic agonists and Sytox Green fluorescence intensities were then measured at 4 h by using a fluorescence plate reader. (**A**) Effects of belinostat and panobinostat on baseline NETosis. (**B**,**C**) Neutrophils were activated with PMA (**B**) or LPS (**C**) in the presence or absence of belinostat or panobinostat. (**D**,**E**) Neutrophils were activated with A23187 (**D**) or ionomycin (**E**) in the presence or absence of belinostat or panobinostat. The full data spread is indicated with lines and boxes are marked with the mean (+), median and upper and lower interquartile ranges. * *p* < 0.05 (One-Way ANOVA with Dunnett post-test, *n* = 5–7). See Supplementary Figures S4 and S5 for additional information.

We next questioned whether increasing concentrations of HDACis have the potential to increase NETosis. To examine this point, we treated neutrophils with belinostat and panobinostat and then evaluated the % NETosis (% of total DNA) by measuring the Sytox Green-stained DNA. The rationale behind using Sytox Green is that it can detect the extracellular DNA, as this dye is impermeable to the cell membrane. Treating neutrophils with 0.5 µM belinostat showed a significant increase of Sytox Green accessible DNA over the 4 h period (Figure 3A; Supplementary Figures S3–S5). However, increased concentrations of belinostat resulted in a gradual decrease of Sytox Green, where the presence of either 20 or 40 µM belinostat significantly inhibited NETosis. The second HDACi, panobinostat, had similar results when Sytox Green assays were performed where a gradual decrease in NETosis was noted. At 6.4 µM panobinostat, NETosis was significantly inhibited when compared to the control (Figure 3A; Supplementary Figure S5).

**Figure 4:**
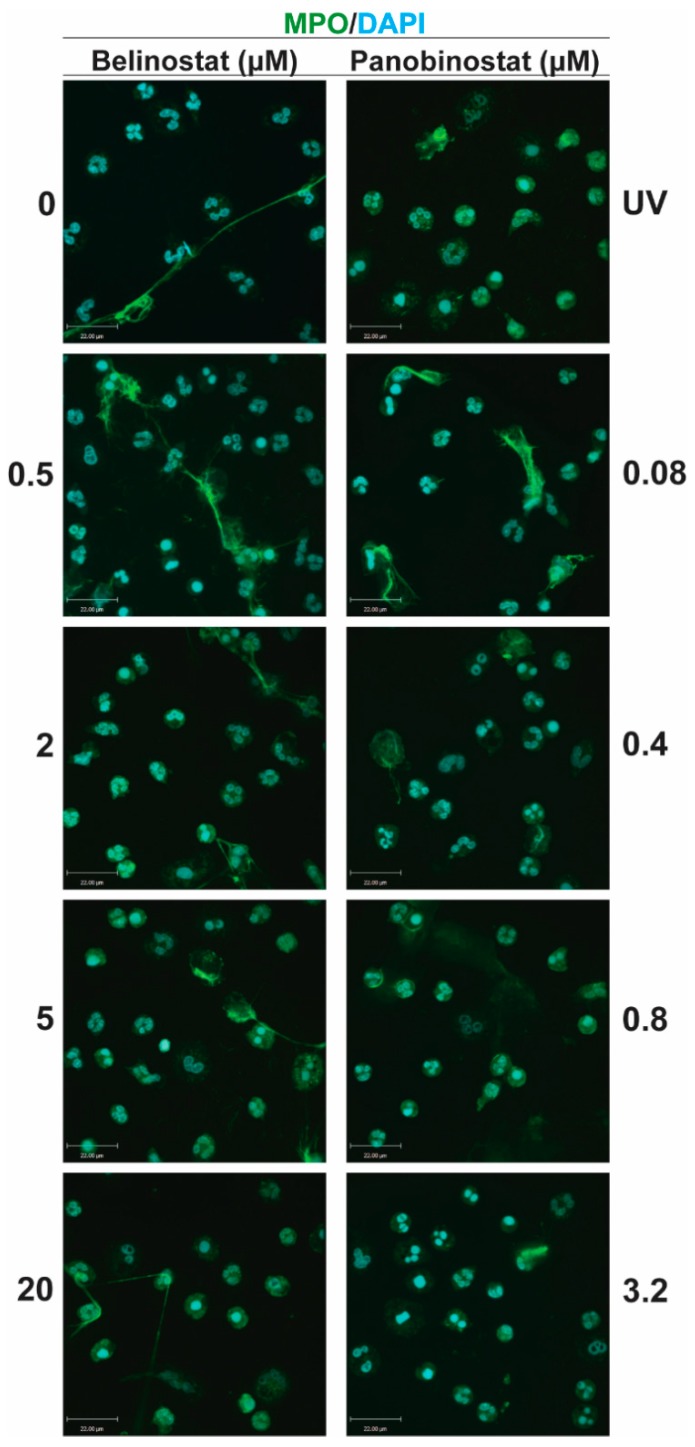
Confocal microscopy images confirm that HDACis inhibit baseline NETosis. Neutrophils were treated with negative control (RPMI), positive control (UV; 0.24 J/cm^2^) or HDACis (0.5, 2, 5 and 20 µM belinostat; 0.08, 0.4, 0.8 and 3.2 µM panobinostat) for 120 min. Cells were then imaged after being fixed and immunostained for myeloperoxidase (MPO) and DNA (DAPI). Neutrophils treated with RPMI show typical polymorphonuclear morphology of neutrophils. When treated with 0.5 µM belinostat or 0.08 µM panobinostat, neutrophils show increased levels of NETosis. However, cells treated with increased concentrations of HDACis decreased MPO colocalization with DNA and increased number of condensed neutrophils. Blue: DAPI staining for DNA; Green: MPO. Scale bar, 14 µm. *n* = 3. See Figure 5 for quantification data and Supplementary Figure S3 for lower magnification confocal imaging.

**Figure 5:**
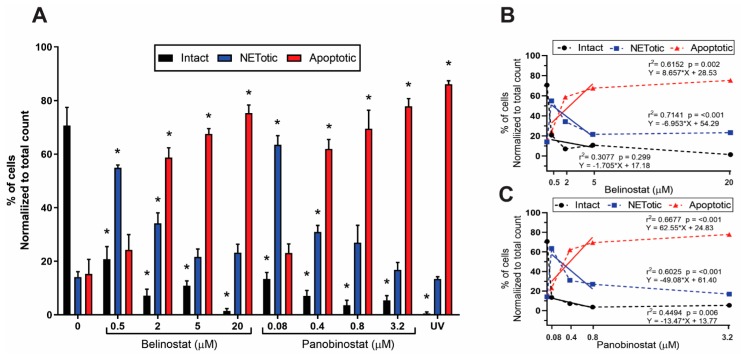
Quantified immunofluorescence images confirm that HDACis have increased cytotoxic effects on neutrophils and is negatively correlated with NETosis. Confocal microscopy images of neutrophils treated with negative control (RPMI), positive control (UV; 0.24 J/cm^2^) or increasing concentrations of HDACis for 120 min were quantified by counting cells stained for DNA and MPO. (**A**) 0.5 µM belinostat or 0.08 µM panobinostat show increased levels of NETosis. However, increasing concentrations of HDAC inhibitors (2, 5 and 20 µM belinostat; 0.4, 0.8 and 3.2 µM panobinostat) show a dose-dependent decrease of NETosis and intact neutrophils but a significant increase in apoptotic cells. (**B,C**) Regression analysis show that apoptosis is positively correlated with increasing concentrations of HDACis but negatively correlated with intact and NETotic cells. The equations of regression lines, r^2^ values and *p*-values are indicated on each panel. Neutrophil counts for each condition were then normalized to the total cell counts. * *p* < 0.05 (Two-Way ANOVA with Dunnett post-test, *n* = 3).

To verify the Sytox Green data, we stained for DNA with DAPI and performed immunofluorescence assays by staining neutrophils against MPO, a known marker of NETs [7]. Images show increased DNA colocalization with MPO when neutrophils were treated with 0.5 µM belinostat or 0.08 µM panobinostat (Figure 4), compared to the control. However, immunofluorescence images show decreased DNA colocalization with MPO when neutrophils were treated with increasing concentrations of HDACis. We next quantified the different types of cells present in each specimen. The image analyses showed a dose-dependent decrease in intact cells when neutrophils were incubated with HDACis (Figure 5A). At the same time, HDAC-treated neutrophils resulted in a decrease of NETotic cells in a dose-dependent manner, except for the 0.5 µM belinostat and 0.08 µM panobinostat conditions, respectively.

### 3.3. Increasing Concentrations of HDAC Inhibitors Promote Apoptosis

To check whether increasing concentrations of HDACis promote other forms of neutrophil death, we examined the nuclear morphology of neutrophils, as nuclei condensation is one of the hallmarks of apoptosis [23]. We also used neutrophils treated with 0.24 J/cm^2^ ultraviolet irradiation which was previously shown to result in apoptotic nuclear morphology without inducing NETosis [24]. Immunofluorescence images showed that cells treated with higher concentrations of HDACis become apoptotic, instead of NETotic (Figure 1 and Figure 4). To verify this observation, we quantified the images and observed a dose-dependent decrease in the proportion of intact cells and increase in apoptotic cells, when neutrophils were treated with increasing concentrations of HDACis (Figure 5A). Regression analyses showed that increasing concentrations of both belinostat and panobinostat result in a significant increase in neutrophils undergoing apoptosis with the reduction of intact and/or NETotic neutrophils (Figure 5B). This effect plateaued at higher concentrations.

**Figure 6:**
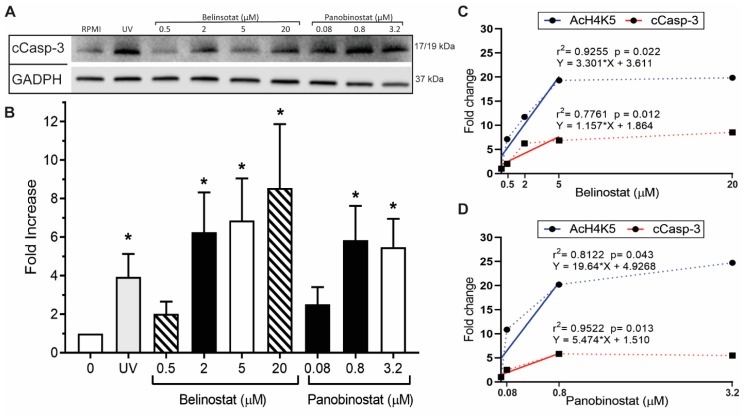
Western blots confirm that HDAC inhibitors induce apoptosis in neutrophils. (**A**) Neutrophils were treated with RPMI (negative control), positive control (ultraviolet (UV); 0.24 J/cm^2^) or HDAC inhibitors (0.5, 2, 5 and 20 µM belinostat; 0.08, 0.8 and 3.2 µM panobinostat) for 90 min. An equal amount of lysates from each condition were separated by polyacrylamide gels, transferred onto a membrane and specific proteins were immunodetected (GADPH for loading control and cCasp-3 for apoptosis). (**B**) The densitometry analyses show a dose-dependent increase in apoptosis when neutrophils were treated with HDAC inhibitors. The values were normalized to the negative control values in each experiment. (**C,D**) Regression analysis of histone acetylation determined by Western blots (AcH4K5, Figure 2; cCasp-3, Figure 6) show that HDACis induce histone acetylation and apoptosis at a significant rate when neutrophils were treated with belinostat (**C**) or panobinostat (**D**) at concentrations of ≤5 µM or ≤0.8µM, respectively. AcH4 and cCasp-3 levels plateau when cells are treated with higher concentrations of HDACis. The equations of regression lines, r^2^ values and *p*-values are indicated on each panel. All data are presented as mean ± SEM; *n* = 3-4; *, *p* < 0.05 compared to respective controls. See Supplementary Figure S6 for the full Western blot.

To further confirm our immunofluorescence imaging data, we performed Western blots and checked for a well-known apoptotic marker, cleavage of caspase 3 (cCasp-3). Neutrophils treated with RMPI (control), 0.5 µM belinostat or 0.08 µM panobinostat showed negligible levels of cCasp-3 (Figure 6A,B; see Supplementary Figure S6 for uncropped Western blots). However, Western blotting showed a significant increase in cCasp-3 levels for neutrophils treated with increased concentrations of HDACis. Close examination of the quantitative data showed that 0.5–5 µM belinostat and 0.08–0.8 µM panobinostat resulted in a significant increase in both AcH4 and cCasp-3 levels (Figure 6C,D). Beyond these concentrations, both histone acetylation and apoptosis levels plateaued. Also, correlational analysis revealed similar patterns between AcH4 and cCasp-3 and suggest that histone acetylation and neutrophil apoptosis are correlated (Supplementary Figure S6). Therefore, increasing concentrations of belinostat and panobinostat inhibit NETosis and promote baseline apoptosis. 

### 3.4. Increasing Concentrations of HDAC Inhibitors Suppress NOX-Dependent NETosis

**Figure 7:**
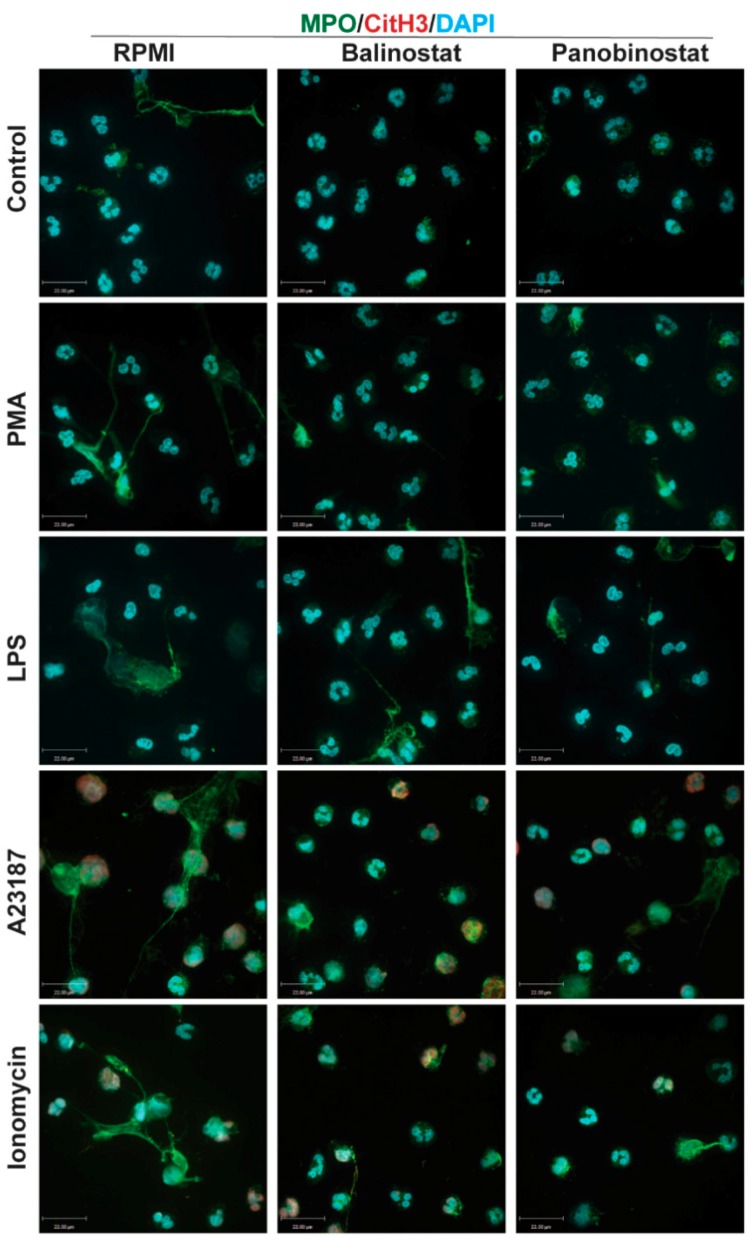
Confocal microscopy images confirm that HDACis promote cytotoxic effects while inhibiting NOX-dependent and -independent NETosis. (A) Neutrophils were treated with negative control (RPMI), NETotic agonists (25 nM PMA; 4 μM A23187; 5 μg/mL LPS from *Escherichia coli* 0128; 5 μM Ionomycin), and/or HDACis (20 µM belinostat; 3.2 µM panobinostat) for 120 min. Cells were then imaged after being fixed and immunostained for myeloperoxidase (MPO), citrullinated histone H3 (CitH3) and DNA (DAPI). Cells treated with RMPI media show typical polymorphonuclear morphology of neutrophils where myeloperoxidase (MPO) can be observed in the cytoplasm. In neutrophils treated with PMA and LPS, MPO co-localizes to NET DNA; limited amounts of CitH3 can be observed. However, CitH3 increases drastically when neutrophils are treated with either A23187 or ionomycin. Blue: DAPI staining for DNA; Green: MPO. Scale bar, 14 µm. *n* = 3.

**Figure 8:**
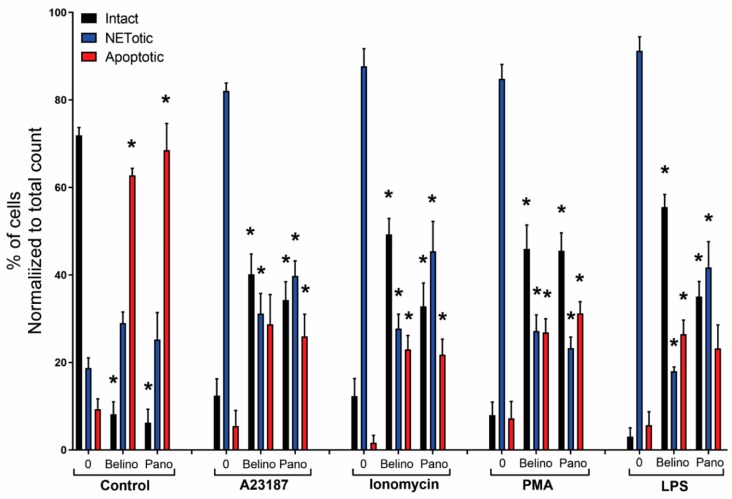
Quantified immunofluorescence images confirm that HDACis promote apoptosis at baseline conditions but prevent neutrophil death in the presence of NETotic agonists. Confocal microscopy images of neutrophils treated with negative control (RPMI), NETotic agonists (25 nM PMA; 4 μM A23187; 5 μg/mL LPS from *E. coli* 0128; 5 μM ionomycin), and/or HDACis (20 µM belinostat; 3.2 µM panobinostat) for 120 min were quantified by counting cells and results showed that cells treated with HDACis have a significant increase in apoptotic cells but a decrease in the total number of intact neutrophils. Interestingly, cells treated with HDACis in presence of NETotic agonists show a significant increase in intact cells but lower apoptotic and NETotic levels when compared to baseline conditions. * *p* < 0.05 (Two-Way ANOVA with Dunnett post-test, *n* = 3).

Two agonists, PMA and LPS, are recognized for their potential to induce NOX-derived ROS in the absence of PAD4 activity [10,25,26]. Also, we have recently shown that histone acetylation in neutrophils, induced by HDACis within the half maximal inhibitory concentration (IC50) concentrations, additively promote NOX-dependent NETosis [12]. Therefore, we questioned whether increasing concentrations of HDACis would have different effects on neutrophils. To check this point, we pre-treated neutrophils with either belinostat or panobinostat and then exposed them to either PMA or LPS for 4 h. The PMA-treated neutrophils had ~40–55% increased levels of NETosis than those treated with RPMI alone (Figure 3B; Supplementary Figure S4). When pre-treated with 0.5 µM belinostat, PMA-induced NETosis significantly increased DNA release by ~20% at 4 h post-treatment (see Supplementary Figure S5B for kinetics graphs). However, when neutrophils were treated with increasing concentrations of belinostat, PMA-induced NETosis was reduced in a time- and concentration-dependent manner, as 10–40 µM belinostat significantly inhibited DNA release by ~30–40%. Similarly, neutrophils pre-treated with panobinostat decreased PMA-induced NETosis in a time- and dose-dependent manner (Figure 3B; Supplementary Figure S5B). Results of the Sytox Green assays for neutrophils activated by HDACis were confirmed by immunofluorescence colocalization of MPO and DNA (Figure 7). We then quantified different types of neutrophil images and results showed that NETotic cells significantly decreased to similar levels as apoptotic cells when neutrophils were cotreated with PMA and HDACis (Figure 8). Yet, intact cells were recorded to be the most abundant in this condition.

Neutrophils treated for 4 h with LPS resulted in a ~25–30% increase in DNA release compared to the control (Figure 3C; Supplementary Figure S4). Also, 0.5–2 µM belinostat and 0.08 µM panobinostat had an additive effect in increasing LPS-induced NETosis by ~10–15%. However, increasing concentrations of HDACis resulted in decreased total DNA release stimulated with LPS in a time- and dose-dependent manner (Supplementary Figure S5C). The NETotic index was ~10–25% lower than LPS-induced NETosis when neutrophils were stimulated with either 10–40 µM belinostat or 0.8–6.4 µM panobinostat. These results were verified with immunofluorescence imaging where HDACis-activated neutrophils showed reduced colocalization of MPO and DNA (Figure 7). In fact, results showed decreased levels of NETotic cells along with increased levels of intact and apoptotic cells (Figure 8). Therefore, increasing concentrations of HDACis have inhibitory effects on NOX-dependent NETosis.

### 3.5. Increasing Concentrations of HDAC Inhibitors Suppress NOX-Independent NETosis

We next questioned whether HDACis have similar inhibitory effects on NOX-independent NETosis. Treating neutrophils with A23187 for 4 h induced NETosis by ~30% above the baseline (Supplementary Figure S4). Pre-incubating cells with 0.5–1 µM belinostat or 0.08 µM panobinostat resulted in significant increase in A23187-induced NETosis (Figure 3D). However, increasing concentrations of HDACis showed a decrease in NETotic index in time- and dose-dependent manner, in which they significantly inhibited NETosis by ~15% when neutrophils were cotreated with 40 µM belinostat or 6.4 µM panobinostat (Supplementary Figure S5D). Results were confirmed by the reduced colocalization of DNA with MPO and CitH3 when we performed immunofluorescence imaging (Figure 7). Quantifying images showed that HDACis significantly reduced the levels of NETotic cells while the levels of intact and apoptotic cells increased significantly (Figure 8). 

Similarly, incubating neutrophils with a well-known NOX-independent agonist, ionomycin, for 4 h resulted in a significant increase in NETosis by ~55–65% when compared with the baseline control (Supplementary Figure S4). The NETotic index slightly increased when neutrophils were treated with ionomycin in the presence of 0.5 µM belinostat or 0.08 µM panobinostat (Figure 3E). On the other hand, ionomycin-induce NETosis reduced in a time- and dose-dependent manner when neutrophils were treated with increasing concentrations of either belinostat or panobinostat (Supplementary Figure S5E). These results were confirmed by performing immunofluorescence imaging where increased levels of intact and apoptotic cells were recorded while the levels of NETotic cells were significantly decreased (Figure 7 and Figure 8). Therefore, increasing concentrations of HDACis suppress NOX-independent NETosis.

### 3.6. Increasing Concentrations of HDAC Inhibitors Promote NOX-, But Not Mitochondria-Derived ROS Production

**Figure 9:**
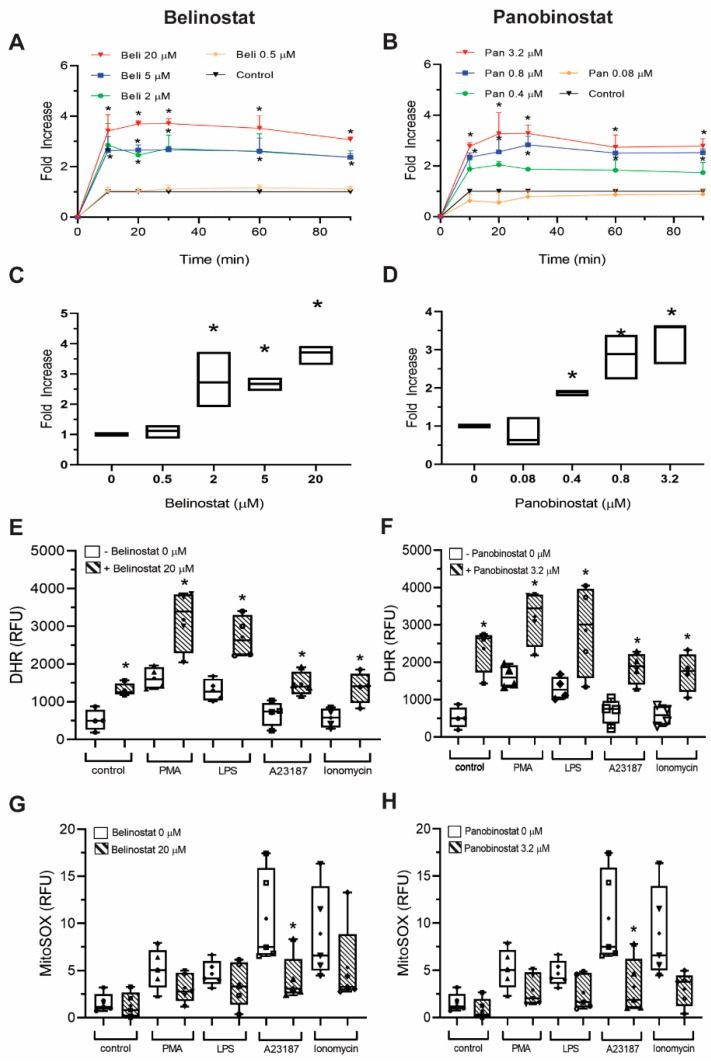
Concentrations of HDACis induce cytosolic but not mitochondrial ROS production. (**A–D**) DHR123 assays measuring NOX-derived ROS production of neutrophils treated with HDACis (0.5, 2, 5 and 20 µM belinostat; 0.08, 0.4, 0.8 and 3.2 µM panobinostat) every 10 min for up to 90 min by using a fluorescence plate reader. Neutrophils treated with belinostat (**A,C**) or panobinostat (**B,D**) show a dose-dependent increase of cytosolic ROS levels. (**C–D**) DHR123 assays at 30 min post-HDACis treatment. (**E–H)** DHR123 and MitoSOX assays measuring NOX- and mitochondrial-derived ROS production of neutrophils treated with HDACis (20 µM belinostat; 3.2 µM panobinostat) at 30 min, respectively. Neutrophils treated with belinostat (**E,G**) or panobinostat (**F,H**) promote intracellular ROS, but not mROS levels. ROS generation data assessed by using DHR123 (**A–F**), while by MitoSOX. (**G,H**) Kinetics data are presented as mean ± SEM (Two-Way ANOVA with Dunnett post-test; *n* = 3). In box graphs, full data spread is indicated with lines and boxes are marked with the mean (+), median and upper and lower interquartile ranges. *, *p* < 0.05 (One-Way ANOVA with Tukey post-test conducted at each time points; *n* = 3, 4). See Supplementary Figures S7 and S8 for additional information.

We have previously reported that both NOX- and mitochondrial-derived ROS are not required to induce HDACi-mediated NETosis [12]. We next questioned whether similar observations would be seen when neutrophils are activated with increasing concentrations of HDACis. To check this point, we treated neutrophils with HDACis for 90 min and used DHR123 probe to measure the ROS produced by NOX, as the probe gets activated once oxidized by ROS [6,26]. When cells were pre-treated with 0.5 µM belinostat or 0.08 µM panobinostat, which were shown to induce NETosis, the cytosolic ROS levels showed similar levels to the baseline control (Figure 9A–D). However, treating neutrophils with increasing concentrations of HDACis had significantly higher cytosolic ROS levels compared to the controls. 

To verify this point, we used two NOX-dependent agonists— PMA and LPS —which are well known to induce NOX-derived ROS production [11]. As expected, DHR123 assays indicated that PMA- and LPS-activated neutrophils had significantly higher cytosolic ROS levels when compared to the control (Supplementary Figure S7). When neutrophils were treated with A23187 or ionomycin for 90 min, cytosolic ROS levels were lower than the control as expected. When we treated neutrophils with HDACis in the presence of PMA or LPS, the cytosolic ROS levels were significantly higher than their corresponding controls (Figure 8E,F; Supplementary Figure S8B,C). Similar observations were made even when cells were cotreated with HDACis and A23187 or ionomycin (Supplementary Figure S8D,E). Note that the cytosolic ROS levels of HDACis-activated neutrophils were similar or higher to those of PMA- and LPS-mediated ROS levels. 

We next questioned whether increasing concentrations of HDACis alter mROS levels. Therefore, we performed MitoSOX assays as the dye fluoresces when it is oxidized by superoxide anion of mitochondrial origin [6]. We treated neutrophils with either A23187 or ionomycin for 90 min and the results showed a significant increase in mROS levels when compared to the control (Supplementary Figure S7B). As expected, PMA- or LPS-activated neutrophils showed an insignificant increase in mROS levels. When we treated neutrophils with HDACis, mROS levels were similar or lower than the baseline control (Figure 9G,F; Supplementary Figure S8A). MitoSOX results also showed an insignificant increase in RFU readings when neutrophils were treated with HDACis in the presence of all NETotic agonists (Supplementary Figure S8B–E). Therefore, higher concentrations of HDACis increase cytosolic ROS production, which is correlated with increased apoptosis.

## 4. Discussion

Several studies have indicated the important role of histone modification in mediating NET formation. Our previous studies showed that citrullination of histone H3 plays a role in unwinding the chromatin exclusively during calcium-mediated NOX-independent NETosis [7,10]. We have recently demonstrated the importance of histone acetylation in NETosis, as belinostat and panobinostat within the IC50 concentrations were able to promote both types of NET formation [12]. Yet, the effect of increasing concentrations of belinostat and panobinostat on neutrophils are still not clearly understood. In this study, we showed that histone hyperacetylation is found in both NETotic and apoptotic cells that are treated with HDACis, either with belinostat or panobinostat (Figure 1 and Figure 2). Increasing higher concentrations of both belinostat and panobinostat significantly inhibited spontaneous, NOX-dependent and -independent NETosis (Figure 3, Figure 4 and Figure 5; Supplementary Figures S3 and S4). Also, we demonstrated that HDACis significantly increased apoptosis levels at baseline (Figure 5, Figure 6 and Figure 7). However, HDACis-treated neutrophils resulted in a significant decrease in intact cells, while similar levels of cells undergoing NETosis were observed when compared to the control. On the other hand, the presence of NETotic agonists partially prevented HDACis-mediated cell death as the number of intact neutrophils significantly increased while apoptotic and NETotic levels were lower than neutrophils treated only with HDAC is (Figure 8). In addition, our results show that HDACis significantly increased NOX— but not mitochondrial—derived ROS production (Figure 9; Supplementary Figures S6 and S7). Collectively, these data show that treating human neutrophils with increasing concentrations of belinostat and panobinostat inhibit NETosis and promote apoptotic effects but prevent cell death in the presence of NETotic agonists.

We recently provided evidence that HDACis have the potential to promote histone acetylation in neutrophils [12]. Also, previous studies have reported that HDACis—for example, SAHA and belinostat—induces AcH4 in oligodendrocyte precursor cells (OPC) and in human promyelocytic leukaemia NB4 and HL-60 cells [27,28]. By performing immunofluorescence imaging and Western blotting, we found consistent results suggesting that belinostat and panobinostat significantly induce histone acetylation in a dose-dependent manner (Figure 1 and Figure 2). We previously showed that HDACis promote AcH4 in neutrophils in both types of NETosis [12]. Therefore, our findings suggest that increasing doses of belinostat and panobinostat can alter their effects on neutrophils.

To better understand the mechanism, we used two ROS probes, DHR123 and MitoSOX, to measure the cytosolic and mitochondrial ROS, respectively. We previously showed that belinostat and panobinostat, within the IC50 values, induce NETosis without altering intracellular ROS levels [12]. In fact, such a finding is supported by the fact that apoptotic microparticles, which have the potential to stimulate numerous immune reactions, were shown to drive ROS-independent NET formation in systemic lupus erythematosus (SLE) [29]. Our current study demonstrates that increasing doses of belinostat and panobinostat results in altering intracellular ROS levels, as DHR123 assays recorded a dose-dependent.

NOX-derived ROS production increased when HDACis were used in the presence and absence of NETotic agonists (Figure 9; Supplementary Figures S6 and S7). In fact, our data are consistent with the previous studies as it was reported that 10 µM belinostat significantly induced intracellular ROS levels when incubated with pancreatic cancer cells [30]. Another study has shown that belinostat also induces a dose-dependent increase in NOX-derived ROS production in thyroid cancer cells [31]. 

Previous studies also show that HDACis-induced histone hyperacetylation results in increased intracellular ROS levels and apoptosis. Dincman et al. (2016) studied the cytotoxicity of HDACi on OPC where they demonstrated that SAHA-induced histone acetylation is associated with the decreased viability of OPCs [27]. Another study showed that treating pancreatic cancer cells with 10 µM belinostat resulted in both increased levels of intracellular ROS production and apoptosis [30]. Indeed, our studies showed that increasing concentrations of belinostat and panobinostat result in significant inhibition of NETosis (Figure 3, Figure 4, Figure 5 and Figure 7; Supplementary Figures S3 and S4) and increased levels of cytosolic ROS and apoptosis (Figure 5, Figure 6, Figure 7 and Figure 8). Linear regression analyses demonstrate a positive correlation between AcH4 and apoptosis but negatively between NETosis and apoptosis (Figure 5 and Figure 6). Consistent with previous studies, promyelocytic leukaemia cells showed a significant increase in cell death when treated with increasing concentrations of belinostat [28]. Similarly, Jiang et al. (2012) studied the effects of panobinostat on HL-60 and acute myelogenous leukaemia (AML) primary cells and found that cells became less viable with increasing concentrations of panobinostat [32]. 

A decrease in ~10–40% NET release could have a significant impact on diseases. Previous studies have demonstrated the post-translational modification of NET histones in lupus and found that induced NET release results in increased levels of autoantibodies that target NETs [33,34]. Our studies may be helpful for understanding the correct application behind using HDACis in treating SLE, as our results show that using high doses of belinostat and panobinostat can inhibit NET release. Also, previous studies mentioned above demonstrate the clinical application of using belinostat and panobinostat in managing numerous types of cancers, such as thyroid and pancreatic cancers [30,31]. 

In summary, increasing concentrations of belinostat and panobinostat significantly induce neutrophil death while inhibiting baseline, NOX-dependent and -independent NETosis. These HDACis have the potential to induce histone acetylation in a dose-dependent manner which was positively correlated with apoptosis but negatively with NETosis (at higher concentrations of the drugs). Our results demonstrated that increasing concentrations of HDACis was accompanied with increased cytosolic but not mitochondrial, ROS production. We conclude that belinostat and panobinostat have a biphasic effect on neutrophils; they induce NETosis within the IC50 concentrations but have apoptosis-inducing effects at higher doses. In the presence of NETotic agonists, high concentrations of HDACis prevent neutrophil death. Hence, these points should be considered when interpreting the effect of HDACis on neutrophils death.

## Supplementary Materials

The supplementary materials are available online at https://www.mdpi.com/2218-273X/9/5/184/s1.

## References

[1] Yousefi S., Stojkov D., Germic N., Simon D., Wang X., Benarafa C., Simon H.U. (2019). Untangling “NETosis” from NETs. Eur. J. Immunol..

[2] Sollberger G., Tilley D.O., Zychlinsky A. (2018). Neutrophil Extracellular Traps: The Biology of Chromatin Externalization. Dev. Cell.

[3] Brinkmann V., Reichard U., Goosmann C., Fauler B., Uhlemann Y., Weiss D.S., Weinrauch Y., Zychlinsky A. (2004). Neutrophil Extracellular Traps Kill Bacteria. Science.

[4] Yuen J., Pluthero F.G., Douda D.N., Riedl M., Cherry A., Ulanova M., Kahr W.H.A., Palaniyar N., Licht C. (2016). NETosing neutrophils activate complement both on their own NETs and bacteria via alternative and non-alternative pathways. Front. Immunol..

[5] Takei H., Araki A., Watanabe H., Ichinose A., Sendo F. (1996). Rapid killing of human neutrophils by the potent activator phorbol 12-myristate 13-acetate (PMA) accompanied by changes different from typical apoptosis or necrosis. J. Leukoc. Biol..

[6] Douda D.N., Khan M.A., Grasemann H., Palaniyar N. (2015). SK3 channel and mitochondrial ROS mediate NADPH oxidase-independent NETosis induced by calcium influx. Proc. Natl. Acad. Sci. USA.

[7] Khan M.A., Palaniyar N. (2017). Transcriptional firing helps to drive NETosis. Sci. Rep..

[8] Metzler K.D., Goosmann C., Lubojemska A., Zychlinsky A., Papayannopoulos V. (2014). Myeloperoxidase-containing complex regulates neutrophil elastase release and actin dynamics during NETosis. Cell Rep..

[9] Papayannopoulos V., Metzler K.D., Hakkim A., Zychlinsky A. (2010). Neutrophil elastase and myeloperoxidase regulate the formation of neutrophil extracellular traps. J. Cell Biol..

[10] Li P., Li M., Lindberg M.R., Kennett M.J., Xiong N., Wang Y. (2010). PAD4 is essential for antibacterial innate immunity mediated by neutrophil extracellular traps. J. Exp. Med..

[11] Khan M.A., Farahvash A., Douda D.N., Licht J.C., Grasemann H., Sweezey N., Palaniyar N. (2017). JNK Activation Turns on LPS-And Gram-Negative Bacteria-Induced NADPH Oxidase-Dependent Suicidal NETosis. Sci. Rep..

[12] Hamam H.J., Khan M.A., Palaniyar N. (2019). Histone Acetylation Promotes Neutrophil Extracellular Trap Formation. Biomolecules.

[13] Kankaanranta H., Janka-Junttila M., Ilmarinen-Salo P., Ito K., Jalonen U., Ito M., Adcock I.M., Moilanen E., Zhang X. (2010). Histone deacetylase inhibitors induce apoptosis in human eosinophils and neutrophils. J. Inflamm..

[14] Johnstone R.W., Licht J.D. (2003). Histone deacetylase inhibitors in cancer therapy: Is transcription the primary target?. Cancer Cell.

[15] Cashen A., Juckett M., Jumonville A., Litzow M., Flynn P.J., Eckardt J., LaPlant B., Laumann K., Erlichman C., DiPersio J. (2012). Phase II study of the histone deacetylase inhibitor belinostat (PXD101) for the treatment of myelodysplastic syndrome (MDS). Ann. Hematol..

[16] Yoon S., Eom G.H. (2016). HDAC and HDAC Inhibitor: From Cancer to Cardiovascular Diseases. Chonnam Med. J..

[17] Eckschlager T., Plch J., Stiborova M., Hrabeta J. (2017). Histone deacetylase inhibitors as anticancer drugs. Int. J. Mol. Sci..

[18] Mottamal M., Zheng S., Huang T.L., Wang G. (2015). Histone deacetylase inhibitors in clinical studies as templates for new anticancer agents. Molecules.

[19] Hammaker D., Firestein G.S. (2018). Epigenetics of inflammatory arthritis. Curr. Opin. Rheumatol..

[20] Reilly C.M., Regna N., Mishra N. (2011). HDAC inhibition in lupus models. Mol. Med..

[21] Roth S.Y., Denu J.M., Allis C.D. (2001). Histone Acetyltransferases. Annu. Rev. Biochem..

[22] Lu X., Wang L., Yu C., Yu D., Yu G. (2015). Histone Acetylation Modifiers in the Pathogenesis of Alzheimer’s Disease. Front. Cell. Neurosci..

[23] Farrell A.W., Halliday G.M., Lyons J.G. (2011). Chromatin structure following UV-induced DNA damage-repair or death?. Int. J. Mol. Sci..

[24] Azzouz D., Khan M.A., Sweezey N., Palaniyar N. (2018). Two-in-one: UV radiation simultaneously induces apoptosis and NETosis. Cell Death Discov..

[25] Wang Y., Li M., Stadler S., Correll S., Li P., Wang D., Hayama R., Leonelli L., Han H., Grigoryev S.A. (2009). Histone hypercitrullination mediates chromatin decondensation and neutrophil extracellular trap formation. J. Cell Biol..

[26] de Souza C.N., Breda L.C.D., Khan M.A., de Almeida S.R., Câmara N.O.S., Sweezey N., Palaniyar N. (2018). Alkaline pH promotes NADPH oxidase-independent neutrophil extracellular trap formation: A matter of mitochondrial reactive oxygen species generation and citrullination and cleavage of histone. Front. Immunol..

[27] Dincman T.A., Beare J.E., Ohri S.S., Gallo V., Hetman M., Whittemore S.R. (2016). Histone deacetylase inhibition is cytotoxic to oligodendrocyte precursor cells in vitro and in vivo. Int. J. Dev. Neurosci..

[28] Savickiene J., Treigyte G., Valiuliene G., Stirblyte I., Navakauskiene R. (2014). Epigenetic and molecular mechanisms underlying the antileukemic activity of the histone deacetylase inhibitor belinostat in human acute promyelocytic leukemia cells. Anticancer. Drugs.

[29] Rother N., Pieterse E., Lubbers J., Hilbrands L., van der Vlag J. (2017). Acetylated histones in apoptotic microparticles drive the formation of neutrophil extracellular traps in active lupus nephritis. Front. Immunol..

[30] Wang B., Wang X.B., Chen L.Y., Huang L., Dong R. (2013). zen Belinostat-induced apoptosis and growth inhibition in pancreatic cancer cells involve activation of TAK1-AMPK signaling axis. Biochem. Biophys. Res. Commun..

[31] Lin S.F., Lin J.D., Chou T.C., Huang Y.Y., Wong R.J. (2013). Utility of a Histone Deacetylase Inhibitor (PXD101) for Thyroid Cancer Treatment. PLoS ONE.

[32] Jiang X.J., Huang K.K., Yang M., Qiao L., Wang Q., Ye J.Y., Zhou H.S., Yi Z.S., Wu F.Q., Wang Z.X. (2012). Synergistic effect of panobinostat and bortezomib on chemoresistant acute myelogenous leukemia cells via AKT and NF-κB pathways. Cancer Lett..

[33] Liu C.L., Tangsombatvisit S., Rosenberg J.M., Mandelbaum G., Gillespie E.C., Gozani O.P., Alizadeh A.A., Utz P.J. (2012). Specific post-translational histone modifications of neutrophil extracellular traps as immunogens and potential targets of lupus autoantibodies. Arthritis Res. Ther..

[34] Pieterse E., Hofstra J., Berden J., Herrmann M., Dieker J., van der Vlag J. (2015). Acetylated histones contribute to the immunostimulatory potential of neutrophil extracellular traps in systemic lupus erythematosus. Clin. Exp. Immunol..

