# Supplementary material for: Histone Deacetylase Inhibitors Dose-Dependently Switch Neutrophil Death from NETosis to Apoptosis
Source: Biomolecules. 2019 May 11;9(5):184. doi: 10.3390/biom9050184 (PMC6571947; doi:10.3390/biom9050184)
Supplement: Supplementary file 1 [file biomolecules-09-00184-s001.pdf]

biomolecules

Supplementary Information

Histone Deacetylase Inhibitors Dose-Dependently Switch Neutrophil Death from NETosis to Apoptosis

Hussein J. Hamam [1,2] and Nades Palaniyar [1,2,3,*]

[1] Program in Translational Medicine, Peter Gilgan Centre for Research and Learning, The Hospital for Sick Children, Toronto, ON, M5G 0A4, Canada; hussein.hamam@sickkids.ca (H.J.H)
[2] Department of Laboratory Medicine and Pathobiology, Faculty of Medicine, University of Toronto, Toronto, ON, M5S 1A8, Canada
[3] Institute of Medical Sciences, Faculty of Medicine, University of Toronto, Toronto, ON, M5S 1A8, Canada
* Correspondence: nades.palaniyar@sickkids.ca

Received: date; Accepted: date ; Published: date

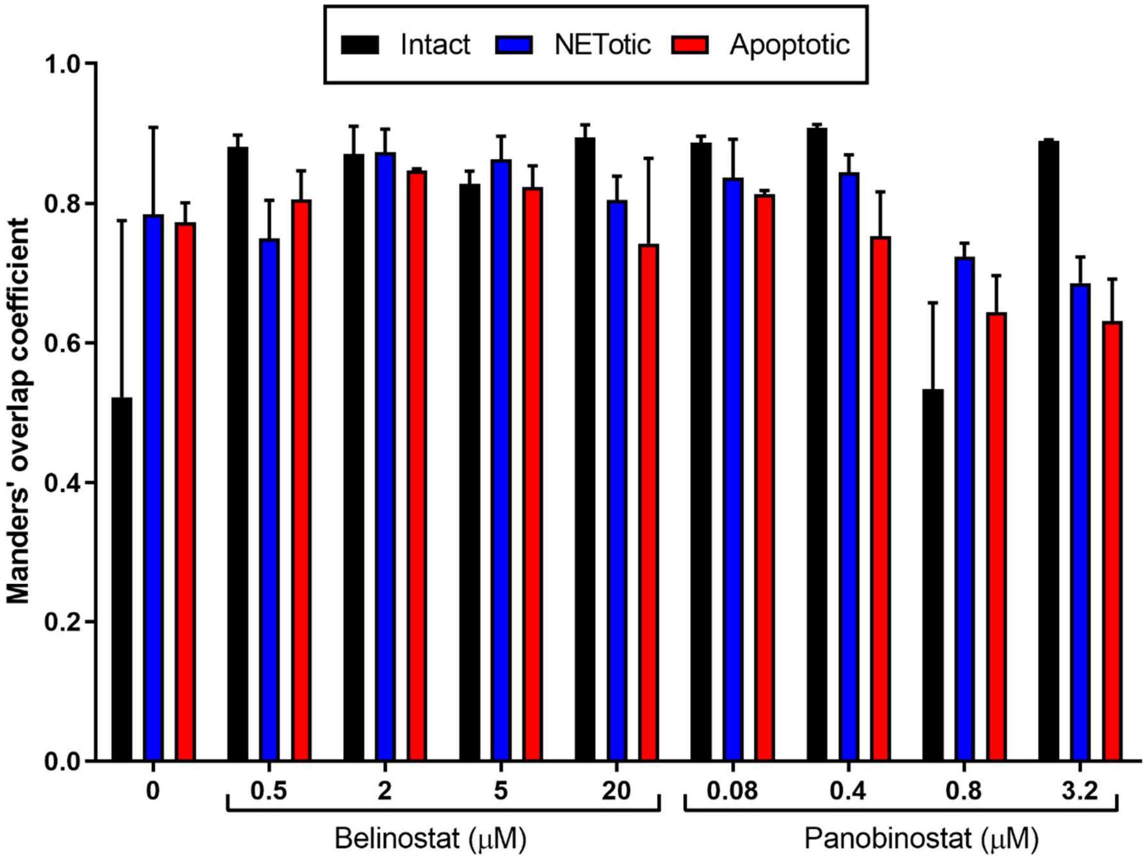

Figure S1. Quantified immunofluorescence images confirm that AcH4 colocalization with DNA. Confocal microscopy images of neutrophils treated with negative control (RPMI) or increasing concentrations of HDACis (2, 5, and 20 μM belinostat; 0.08, 0.4, 0.8, and 3.2 μM panobinostat) for 120 min were analyzed for Manders' overlap coefficient between H4K5ac and DAPI (DNA). Data show that similar overlap coefficients were similar between intact, NETotic and apoptotic cells in all experimental conditions, suggesting that AcH4 overlapped with DAPI. $n = 3$.

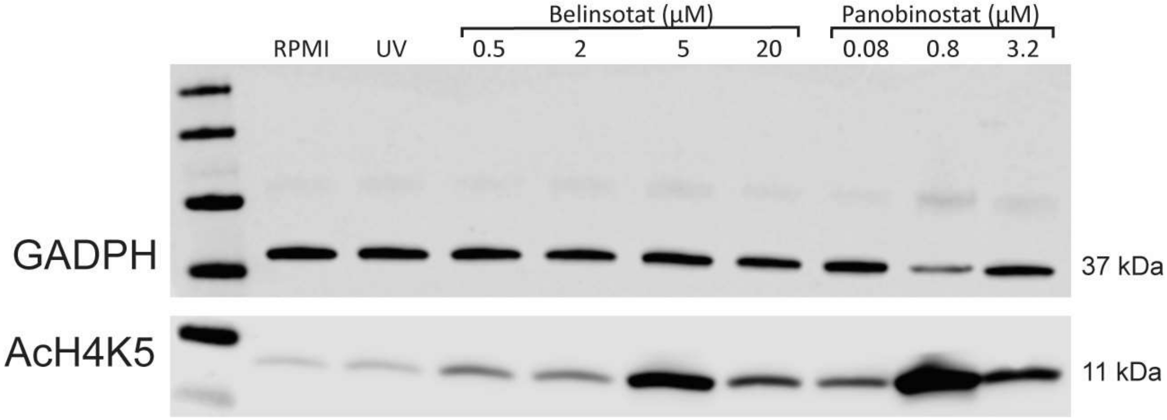

Figure S2. Western blots showing that HDAC inhibitors induce histone acetylation. Neutrophils were treated with RPMI (negative control), or HDAC inhibitors (0.5, 2, 5, and 20 μM belinostat; 0.08, 0.8, and 3.2 μM panobinostat) for 90 min. RIPA buffer and DNAse were used, along with sonication, to prepare whole cell lysates. For each condition, 25 μg/ml lysates were separated by using 4-20% precast protein gels. Overnight staining for GADPH (loading control) and histone acetylation (H4K5ac) show increased histone acetylation when neutrophils are treated with HDAC inhibitors. Image is representative of 3-4 independent experiments. See Figure 2 for densitometry analysis.

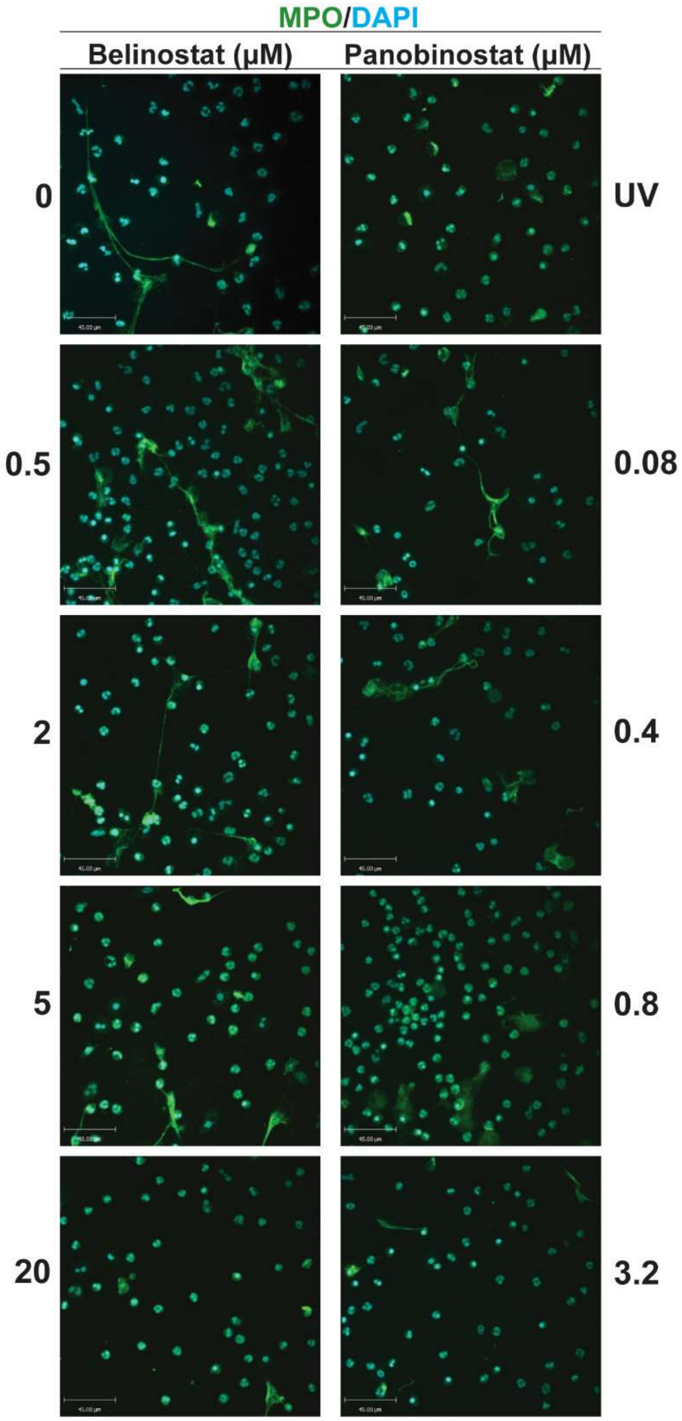

Figure S3. Lower magnification confocal microscopy images confirm that HDACis inhibit baseline NETosis. Neutrophils were treated with negative control (RPMI), positive control (UV; 0.24 J/cm^2), or HDACis (0.5, 2, 5, and 20 µM belinostat; 0.08, 0.4, 0.8, and 3.2 µM panobinostat) for 120 min. Cells were then imaged after being fixed and immunostained for myeloperoxidase (MPO) and DNA (DAPI). Neutrophils treated with RPMI show typical polymorphonuclear morphology of neutrophils. When treated with 0.5 µM belinostat or 0.08 µM panobinostat, neutrophils show increased levels of NETosis. However, cells treated with increased concentrations of HDACis decreased MPO colocalization with DNA and increased number of condensed neutrophils. Blue: DAPI staining for DNA; Green: MPO. Scale bar, 45 µm. n = 2-3.

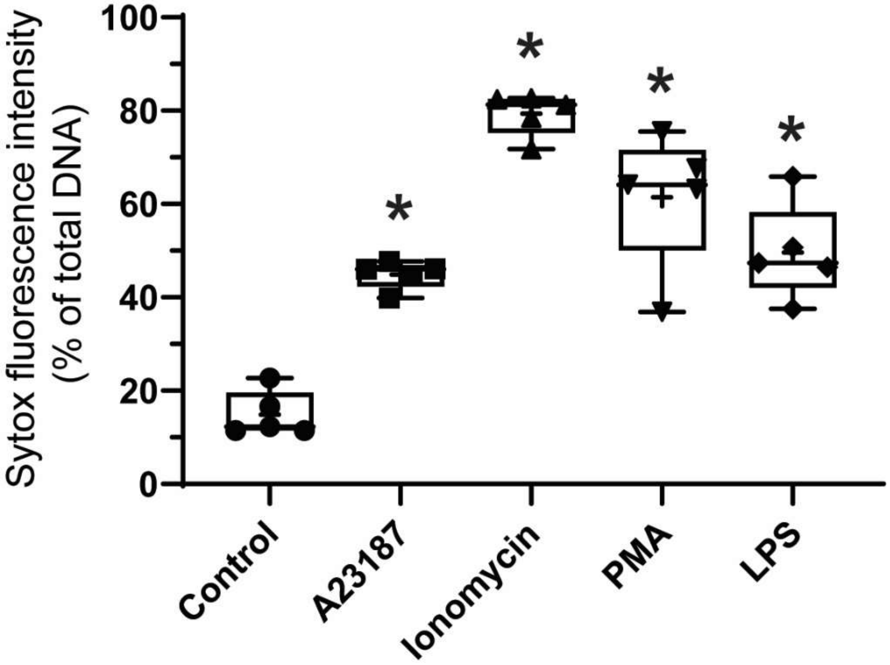

Figure S4. Sytox Green assays comparing the levels of NETosis of PMA, LPS, A23187 and ionomycin with the baseline. Neutrophils were treated with NETotic agonists (25 nM PMA; 4 µM A23187; 5 µg/ml LPS from *E. coli* 0128; 5 µM Ionomycin) and Sytox Green fluorescence intensities were then measured every 60 min for up to 4 h by using a fluorescence plate reader. Cells treated with all NETotic agonists show a significant increase in NETotic index at 240 min post-treatment, where ionomycin-induced cells recorded the highest NETotic index. PMA- and LPS-stimulated neutrophils showed higher levels of NET formation when compared to that of A23187. All data are presented as mean ± SEM; *, $p < 0.05$ (One-Way ANOVA with Dunnett post-test; n = 5-7).

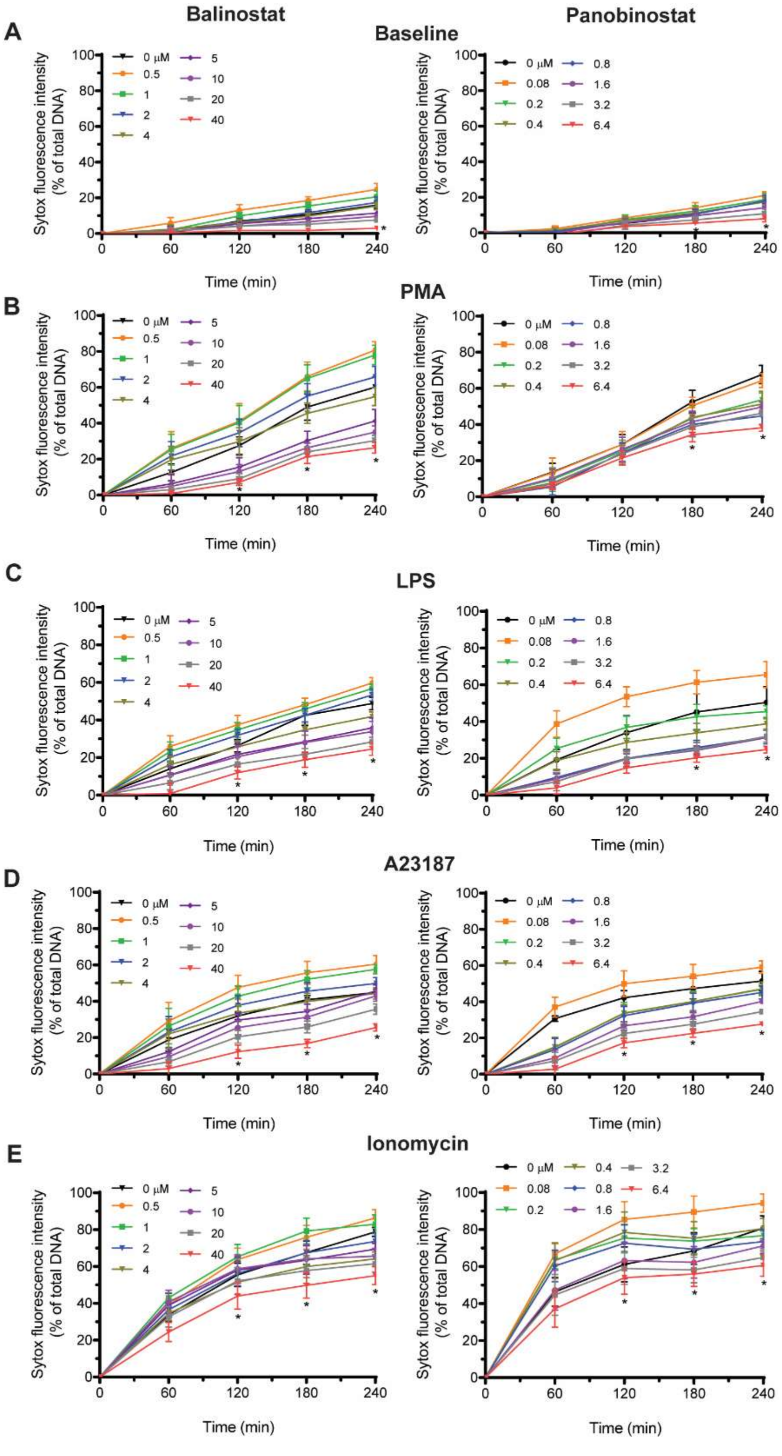

Figure S5. Sytox Green assays suggest that belinostat and panobinostat inhibit baseline NETosis as well as both NOX-dependent and -independent NETosis. Neutrophils were treated with HDACis and/or NETotic agonists (25 nM PMA; 4 μM A23187; 5 μg/ml LPS from *E. coli* 0128; 5 μM Ionomycin), and Sytox Green fluorescence intensities were then measured every 60 min for up to 4 h by using a fluorescence plate reader. (**A-E)** Cells treated with 0.5 μM belinostat or 0.08 μM panobinostat show increased NETotic index for cells treated with control (**A**), PMA (**B**), LPS (**C**), A23187 (**D**), or ionomycin (**E**). However, increased concentrations of HDACis showed decreased levels of NETosis in a dose-dependent manner. All data are presented as mean ± SEM; *, $p < 0.05$ (Two-Way ANOVA with Dunnett post-test; n = 5-7).

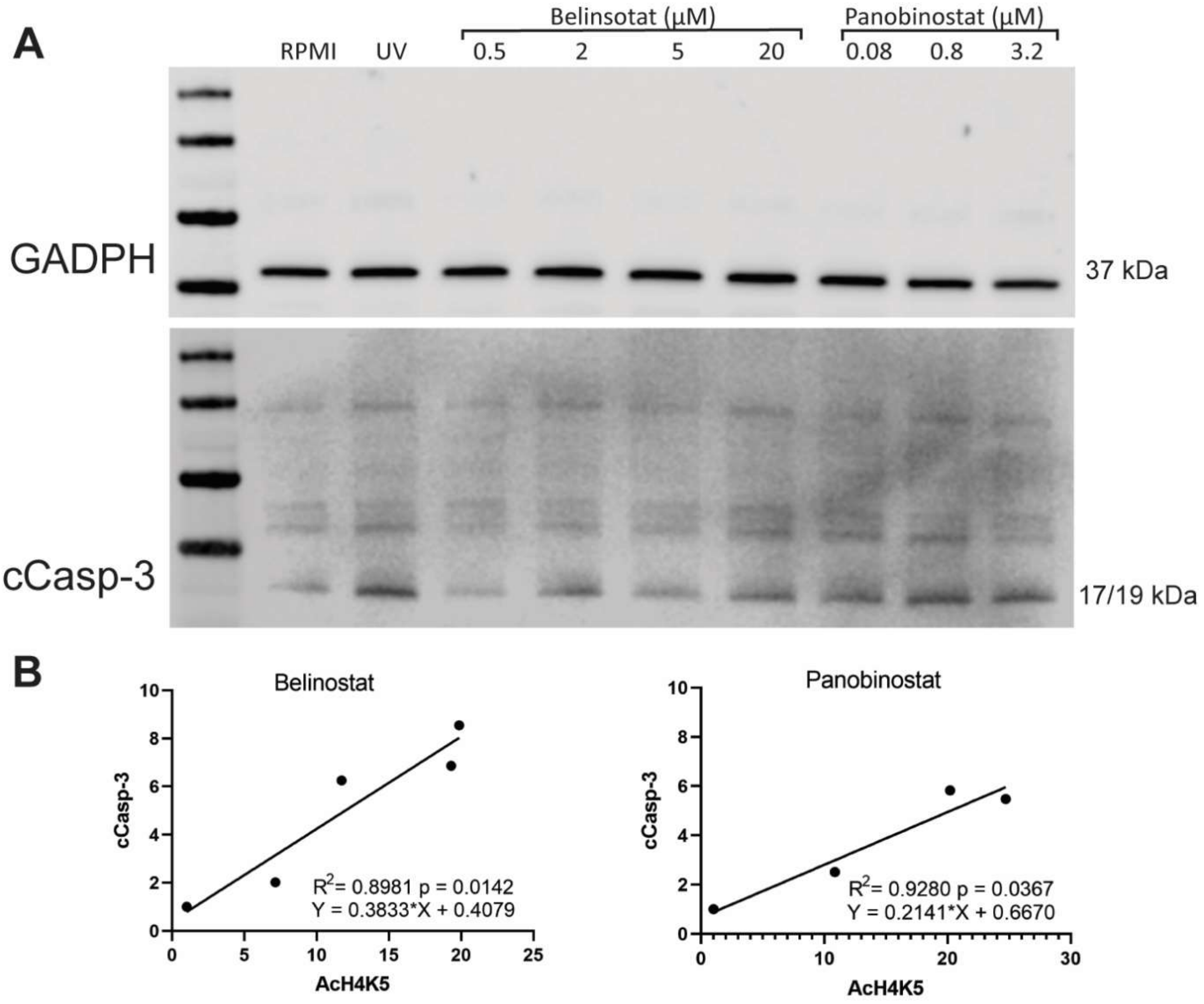

Figure S6. Western blots confirm that HDAC inhibitors induce cytotoxic effects on neutrophils. (**A**) Neutrophils were treated with RPMI (negative control), positive control (UV; 0.24 J/cm^2), or HDAC inhibitors (0.5, 2, 5, and 20 µM belinostat; 0.08, 0.8, and 3.2 µM panobinostat) for 90 min. RIPA buffer and DNAse were used, along with sonication, to prepare whole cell lysates. For each condition, 25 µg/ml lysates were separated by using 4-20% precast protein gels. Overnight staining for GADPH (loading control) and cleaved caspase 3 (cCasp-3) show increased levels of apoptosis when neutrophils are treated with HDAC inhibitors. Image is representative of 3-4 independent experiments. (**B**) Correlational analysis shows positive correlation between histone acetylation and neutrophil apoptosis, which were determined by Western blots (AcH4K5, Figure 2; cCasp-3, Figure 6), for neutrophils treated with either belinostat or panobinostat. See Figure 6 for densitometry analysis.

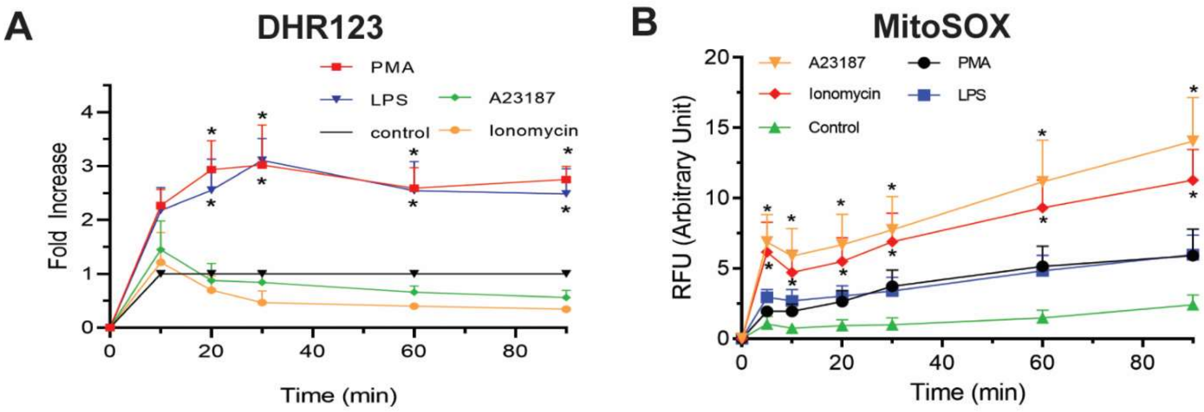

Figure S7. DHR123 and MitoSOX assays for neutrophils treated with NOX-dependent and -independent agonists. (A) DHR123 assays measuring NOX-mediated ROS production of neutrophils. (B) MitoSOX assay measuring mROS levels. Cells treated with PMA or LPS have increased levels of cytosolic ROS, but not mROS. Induced levels of mROS are found for neutrophils treated with A23187 or ionomycin. All data are presented as mean ± SEM; *, $p < 0.05$ (Two-Way ANOVA with Dunnett post-test; n = 3).

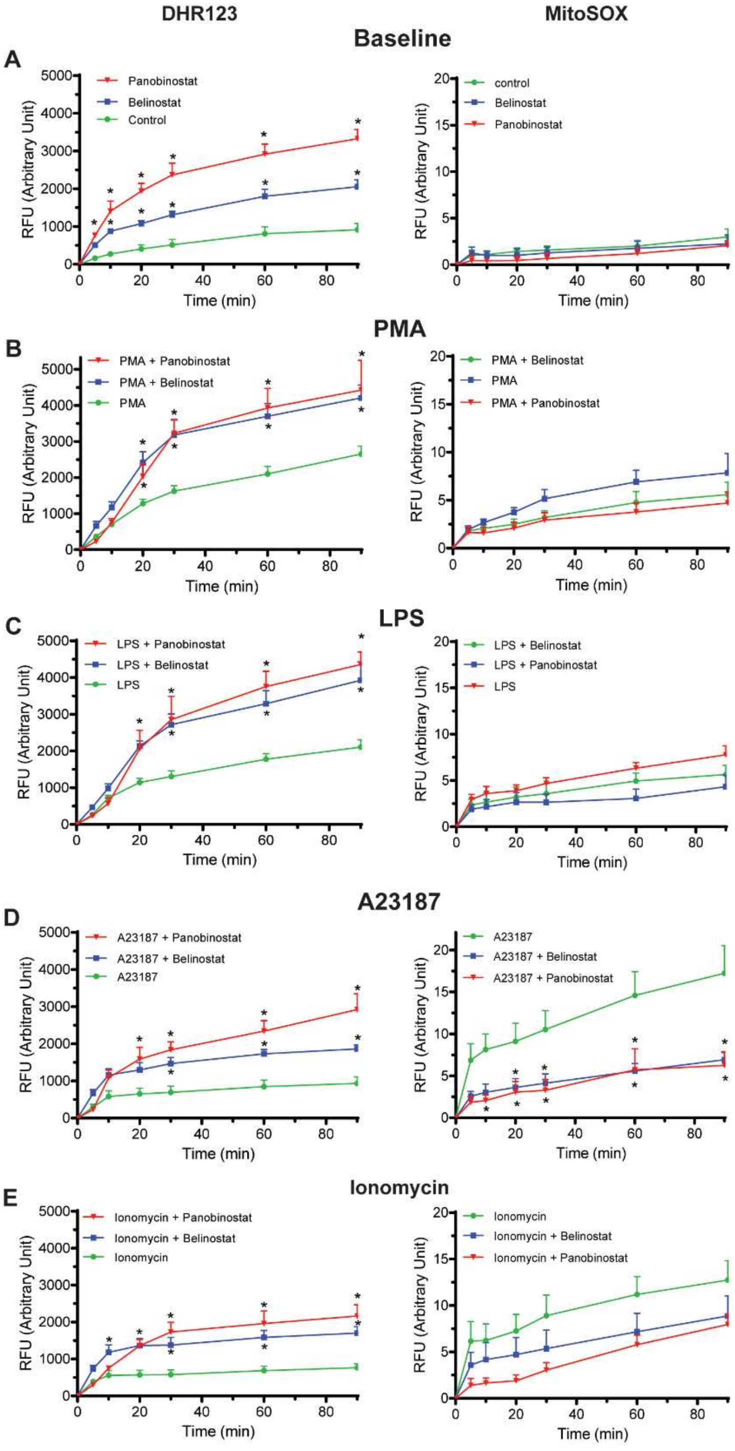

Figure S8. Increasing concentrations of HDACis induce cytosolic, but not mitochondrial ROS production. DHR123 assays measuring NOX-mediated ROS production of neutrophils. MitoSOX assay measuring mROS levels. (A) Neutrophils treated with belinostat (20 μM) or panobinostat (3.2 μM) have increased levels of cytosolic, but not mitochondrial-ROS. (B, C) Cells treated with PMA (B) or LPS (C) have increased levels of cytosolic ROS, but not mROS. (D, E) Induced levels of mROS are found for neutrophils treated with A23187 (D) or ionomycin (E). Neutrophils treated with HDACis in the presence of NETotic agonists show increased levels of NOX-derived ROS, but not mROS. All data are presented as mean ± SEM; *, $p < 0.05$ (Two-Way ANOVA with Dunnett post-test; n = 4-5).
